# Rapid Breathing as a Pain Obtunder in Minor Surgery, Obstetrics, the General Practice of Medicine, and of Dentistry

**Published:** 1881-06

**Authors:** W. G. A. Bonwill


### RAPID BREATHING AS A PAIN OBTUNDER IN MINOR SURGERY, OBSTETRICS, THE GENERAL PRACTICE OF MEDICINE AND OF DENTISTRY.

BY W. G. A. BONWILL, D.D.S.

*Read before the Philadelphia County Medical Society, May,* 1880.

Through the kind invitation of your Directors, I am present to give you the history of "rapid breathing" as an analgesic agent, as well as my experience therein since I first discovered it. It is with no little feeling of modesty that I appear before such a learned and honorable body of physicians and surgeons, and I accept the privilege as a high compliment. I trust the same liberal spirit which prompted you to call this subject to the light of investigation will not forsake you when you have heard all I have to say and you sit in judgment thereon. Sufficient time has now elapsed since the first promulgation of the subject for the shafts of ridicule to be well nigh spent (which is the common logic used to crush out all new ideas), and it is to be expected that gentlemen will look upon it with all the charity of a learned body, and not be too hasty to condemn what they have had but little chance to investigate; and, of course, have not practiced with that success which can only come from an intelligent understanding of its application and *modus operandi.*

Knowing the history of past discoveries, I was well prepared for the crucible. I could not hope to be an exception. But, so far, the medical profession have extended me more favor than I have received at the hands of the dental profession.

My first conception of the analgesic property of a pain obtunder in contradistinction to its anæsthetic effect which finally led to the discovery of the inhalation of common air by " rapid breathing, "

was in 1855, while performing upon my own teeth certain operations which gave me intense pain, and I could not afford to hurt myself, without a resort to ether and chloroform. These agents had been known so short a time that no one was specially familiar with their action. Without knowing whether I could take chloroform administered by myself, and at the same time perform with skill the excavation of extremely sensitive dentine or tooth-bone, as if no anæsthetic had been taken, and not be conscious of pain, was more than the experience of medical men at that time could assure me. But, having a love for investigation of the unknown, I prepared myself for the ordeal. By degrees I took the chloroform until I began to feel very plainly its primary effects, and knowing that I must soon be unconscious, I applied the excavator to the carious tooth, and, to my surprise, found no pain whatever, but the sense of touch and hearing were marvelously intensified. The small cavity seemed as large as a half bushel; the excavator more the size of an ax; and the sound was equally magnified. That I might not be mistaken, I repeated the operation until I was confident that anæsthetics possessed a power not hitherto known, that of analgesia. To be doubly certain, I gave it in my practice, in many cases with the same happy results, which saved me from the risks incident to the secondary effects of anæsthetics, and which answered for all the purposes of extracting from one to four teeth. Not satisfied with any advance longer than I could find a better plan, I experimented with the galvanic current (to and fro) by so applying the poles that I substituted a stronger impression by electricity from the nerve centers or ganglia to the periphery, than was made from the periphery to the brain. This was so much of a success that I threw aside chloroform and ether, and removed the living nerve of a tooth with instruments instead of using arsenic, and for excavating sensitive caries in teeth, preparatory to filling, as well as many teeth extracted by it. But this was short-lived, for it led to another step. Sometimes I would inflict severe pain in cases of congested pulps or from its hasty application, or pushing it to do too much, when my patient invariably would draw or inhale the breath *very forcibly and rapidly*. I was struck with the repeated coincidence, and was led to exclaim:

"Nature's anæsthetic." This then reminded me of boyhood's bruises. The involuntary action of every one who has a finger hurt is to place it to the mouth and draw violently in the air and hold it for an instant, and again repeat it until the pain is subdued. The same action of the lungs occurs, except more powerfully, in young children who take to crying when hurt. It will be noticed they breathe very rapidly while furiously crying, which soon allays the irritation, and sleep comes as the sequel. Witness also when one is suddenly startled, how violently the breath is taken, which gives relief. The same thing occurs in the lower animals when pain is being inflicted at the hand of man.

This was advance No. 3, and so sure was I of this new discovery, that I at once made an application while removing decay from an extremely sensitive tooth. To be successful, I found I must make the patient take the start, and I would follow with a thrust from the excavator, which move would be accomplished before the lungs could be inflated. This was repeated for at least a minute, until the operation was completed, I always following immediately or synchronously with the inhalation.

This led to step No. 4, which resulted in its application to the extracting of teeth and other operations in minor surgery.

Up to this time I had believed the sole effect of the rapid inhalation was due to mere diversion of the will, and this was the only way nature could so violently exert herself—that of controlling the involuntary action of the lungs to her uses by the *safety valve*, under the voluntary movement.

The constant breathing of the patient for thirty seconds to a minute left him in a condition of body and mind resembling the effects of ether and chloroform in their primary stages. I could but argue that the prolonged breathing each time had done it: and, if so, then there must be some specific effect over and above the mere diversion by the will. To what could it be due? To the air alone, which went in excess into the lungs in the course of a minute! Why did I not then immediately grasp the idea of its broader application as now claimed for it? It was too much, gentlemen, for that hour. Enough had been done in this fourth step of conception to rest in the womb of time, until by evolution a higher step could be made at the maturity of the child. Being self-satisfied with my own baby, I watched and caressed it until it could take care of itself, and my mind was again free for another conception.

The births at first seemed to come at very short intervals: but see how long it was between the fourth and the fifth birth. It was soon after that my mind became involved in inventions—a hereditary outgrowth—and the electric mallet and then the dental engine, the parent of your surgical engine, to be found in the principal hospitals of this city, took such possession of my whole soul, that my air analgesic was left slumbering. It was not until August, 1875—nineteen years after—that it again came up in full force, without any previous warning.

This time it was no law of association that revived it; but it seemed the whispering of some one in the air—some ethereal spirit, if you please—which instituted it, and advanced the following problem: "Nitrous oxide gas is composed of the same elements as ordinary air, with a larger equivalent of oxygen, except it is a chemical compound, not a mechanical mixture, and its anæsthetic effects are said to be due to the excess of oxygen. If this be a fact, then why can you not produce a similar effect by rapid breathing for a minute, more or less, by which a larger quantity of oxygen is presented in the lungs for absorption by the blood?"

This query was soon answered by asking myself another. "If the rapid inhalation of air into the lungs does not increase the heart's action and cause it to drive the blood in exact ratio to the inhalations, then *I can* produce partial anæsthesia from this excess of oxygen brought about by the voluntary movements over the ordinary involuntary action of the lungs." The next question was: "Will my heart be affected by this excess of air in the lungs to such an extent that there will be a full reciprocity between them?" Without making any trial of it, I argued that, while there is no other muscular movement then that of the chest as under the control of the will, and as nature has given to the will the perfect control over the lungs to supply more or less air as is demanded by the pneumogastric nerve for the immediate wants of the economy, when the *involuntary action* is not sufficient; and the heart not being under the control of the will, and its action never accelerated or diminished except by a specific poison, or from the general activity of the person in violent running or working, the blood is forced into the heart faster and it must get rid of it, when a larger supply of oxygen is demanded and rapid breathing must occur, or asphyxia result. I was not long in deciding that the heart *would not be accelerated* but a trifle—say a tenth—and, under the circumstances, I said: "The air *is* an anæsthetic."

From this rapid course of argument, I was so profoundly convinced of its truth, that without having first tried it upon my own person, I would have sat where I was, upon the curbstone, and had a tooth removed, with the perfect expectation of absence of pain and of still being conscious of touch. While yet walking, I commenced to breathe as rapidly as possible, and, as anticipated, found my steps growing shorter and shorter, until I came to a stand, showing to my mind clearly that my argument in advance was right, so far as locomotion was concerned; and, upon referring to my pulse, I found but little acceleration.

To what other conclusion could I arrive from this argument, with the foundation laid nineteen years before, when I established on my own person by experiment the fact of analgesia as induced from chloroform, with the many experiments in rapid respiration on tooth bone?

From this moment until its first application to the extraction of a tooth you can well imagine my suspense. That I might not fail in the very first attempt, I compelled myself and others in my household to breathe rapidly to investigate the phenomenon. This gave me some idea as to the proper method of proceeding in its administration.

The first case soon appeared, and was a perfect success, going far beyond my anticipations, for the effect was such as to produce a partial paralysis of the hands and arms to the elbow. Again and again I tried it in every case of extraction and many other experiments, doubting my own senses for a long time, at a result so anomalous and paradoxical. I was reminded just here of a phenomenon which gave me additional proof—that of blowing a dull fire to revive it. For a minute or so one blows and blows in rapid succession, until, rising from the effort, a sense of giddiness for a few moments so overcomes that the upright position is with difficulty maintained. In this condition you are fitted for having a tooth extracted or an abscess lanced.

Believing that I had something new to offer which might be of use to suffering humanity, I read the first article upon it Nov. 17, 1875, before the Franklin Institute. Shortly after I was invited before the Northern Medical Society of this city to address them thereon. A number of medical gentlemen have been using it in their practice, while the bulk of them have spurned it as "negative" and preposterous, without an effort at trying it, which I can *now* very well understand.

Unless one is aware of the fact that in the use of any agent which has the power to suspend the volition, it can be taken to that point where he is still conscious of *touch and hearing*, and at the same time not cognizant of pain inflicted, the action of rapid breathing can not be understood. And I regret to say that of three-fourths of the medical men I have talked with on the subject they had not been aware of such a possibility from ether and chloroform. Until this analgesic state could be established in their minds it was impossible to convince them that the excess of oxygen, as obtained by rapid breathing, could be made to produce a similar effect. *I* should have been as reluctant as any one to believe it, had I not personally experienced the effect while performing an operation which would otherwise have been very painful. Such a result could not well be reached by any course of reasoning alone.

Has it proven in my practice what has been claimed for it—a substitute for the powerful anæsthetics in minor operations in surgery? Most emphatically, yes! So completely has it fulfilled its humble mission in my office, that I can safely assert there has not been more than five per cent. of failures. I have given it under all circumstances of diseased organs, and have seen no other than the happiest results in its after effects. It may well be asked just here: Why has it not been more generally and widely used by the dental profession as well as the medical, if it is really what is claimed for it? The most satisfactory and charitable answer to be given is, the failure upon their part to comprehend the *fact*, as existing in chloroform and ether, that there is such a state as analgesia; or, in other words, that the animal economy is so organized, while the sense of touch is not destroyed, but rather increased, the mind of the subject fails to perceive a sense of pain when anæsthetics are given, and the effects are manifested in the primary stage. As I before intimated, such is the knowledge possessed by most of those who administer ether and chloroform. This was enough to cause nearly every one to look upon it as a bubble or air castle. Many gentlemen told me they tried it upon themselves, and, while it affected them very seriously by giddiness, they still *retained consciousness*; and, such being the case, no effect could be produced for obtunding pain. Others told me they were afraid to continue the breathing, alarmed at the vertigo induced. And the practitioner who has adopted it more effectively than any other laughed at me when I first told him of the discovery; but his intimate association with me changed his views after much explanation and argument between us.

It was hardly to be expected that without this knowledge of analgesia, and without any explanation from me as to the *modus operandi* of rapid breathing, other than a few suggestions or directions as to how the effect was induced, even the most liberal of medical men should be able to make it effective, or have the least disposition to give it a preliminary trial upon themselves, and, of course, would not attempt it upon a patient. Notwithstanding, it found a few adherents, but only among my personal *medical* friends, with whom I had an opportunity to explain what I believed its physiological action, and the cases of success in my own practice. To this I have submitted as among the inevitable in the calendar of discoveries of all grades.

My own profession have attempted to *ridicule* it out of its birthright and possible existence, which style of argument is not resorted to by true logicians.

To all this I can truly say I have not for one moment faltered.

I could afford to wait. The liberality of this society alone fully compensates for the seeming indisposition of the past, believing that it is proper that every advance should be confronted, and, if in time found worthy, give it God speed.

From its first conception I have diligently labored to solve its *modus operandi*, and the doubt in my own mind as to whether I could be mistaken in my observations. I asked the opinion of our best chemical teachers if air could have such effect. One attributed it to oxygen stimulation, and the other to nitrogen. Another gentleman told me the medical profession had come to the conclusion, that it was possible for me to thus extract teeth, but it was due solely to my strong *personal magnetism*, (which power I was not before aware I possessed).

Now, from what I have related of the successive and natural steps which finally culminated in this process or plan of analgesia induced by an excess of ordinary air taken forcibly into the lungs above what is necessary for life, and from what I shall state as to the apparently anomalous or paradoxical effects, with its physiological action, and the simple tests made upon each of my patients, I shall trust to so convince you of its plausibility and possibility that it will be made use of in hundreds of minor operations where ether and chloroform are now used.

Aside from my assertion and that of its friends, that the effects can be produced by air alone, you must have some light shed upon the causes of its physiological action, which will appeal to your *medical* reason.

To assign an action to any drug is difficult, and in the cases of ether and the other anæsthetics a quarter of a century still finds many conflicting opinions. This being true, you will deal leniently with me for the opinion I hold as to their analgesic action. Of course it will be objected to, for the unseen is, to a great extent, unknowable. Enough for my argument, however; it seems to suit the case very well without looking for another; and while it was based on the phenomenon resulting from many trials, and not the trials upon it as a previous theory, I shall be content with it until a better one can be found.

What is it I claim as a new discovery, and the facts and its philosophy?

I have asserted that I can produce, from rapidly breathing common air at the rate of a hundred respirations a minute, a similar effect to that from ether, chloroform, and nitrous oxide gas, in their primary stages; and I can in this way render patients sufficiently insensible to acute pain from any operation where the time consumed is not over twenty to thirty seconds. While the special senses are in partial action, the sense of pain is obtunded, and in many cases completely annulled, consciousness and general sensibility being preserved.

To accomplish this, each patient must be instructed how to act and what to expect. As simple as it may seem, there is a proper and consistent plan to enable you to reach full success. Before the patient commences to inhale he is informed of the fact that, while he will be unconscious of pain he will know full or partially well every touch upon the person; that the inhalation must be vigorously kept up during the whole operation without for an instant stopping; that the more energetically and steadily he breathes, the more perfect the effect, and that if he cease breathing during the operation, pain will be felt. Fully impress them with this idea, for the very good reason that they may stop when in the midst of an operation, and the fullest effects be lost. It is obligatory to do so on account of its evanescent effects, which demand that the patient be pushed by the operator's own energetic appeals to "go on." It is very dfficult for any person to respire more than one hundred times to the minute, as he will become by that time so exhausted as not to be able to breathe at all, as is evidenced by all who have thus followed my directions. For the next minute following the completion of the operation the subject will not breathe more than once or twice. Very few have force enough left to raise hand or foot. The voluntary muscles have nearly all been subjugated and overcome by the undue effort at forced inhalation of one hundred instead of seventeen, the normal standard. It will be more fully understood further on in my argument why I force patients, and am constantly speaking to them to go on.

I further claim that for the past four years, so satisfactory has been the result of this system in the extracting of teeth and deadening extremely sensitive dentine, there was no longer any necessity for chloroform, ether, or nitrous oxide in the dental office. That such teeth as cannot be extracted by its aid can well be preserved and made useful, except in a very few cases, who will not be forced to breathe.

The anæsthetics, when used in major operations, where time is needed for the operation, can be made more effective by a lesser quantity when given in conjunction with "rapid breathing." Drs. Garrettson and Hewson, who have thus tried it, tell me it takes one-half to three-fourths less, and the after effects are far less nauseating and unpleasant.

As an agent in labor, where an anæsthetic is indicated, it is claimed by one who has employed it (Dr. Hewson) in nearly every case for three years, he has used "rapid breathing" solely, and to the exclusion of chloroform and ether. For this I have his assertion, and have no doubt of it whatever, for if any agent could break down the action of the voluntary muscles of the parts involved, which prevent the involuntary muscles of the uterus from having their fullest effect, it is this. The very act of rapid breathing so affects the muscles of the abdomen as to force the contents of the uterus downward or outward, while the specific effect of the air at the end of a minute's breathing leaves the subject in a semi-prostrate condition, giving the uterus full chance to act in the interim, because free of the will to make any attempt at withholding the involuntary muscles of the uterus from doing their natural work. It is self-evident; and in this agent we claim here a boon of inestimable value. And not least in such cases is, there is no danger of hemorrhage, since the cause of the effect is soon removed.

In attestation of many cases where it has been tried, I have asked the mother. and, in some cases, the attendants, whether anything else had been given, and whether the time was very materially lessened; there has been but one response, and that in its favor.

Gentlemen, if we are not mistaken in this, you will agree with me in saying that it is no mean thing, and should be investigated by intelligent men and reported upon. From my own knowledge of its effects in my practice, I am bound to believe this gentleman's record.

I further claim for it a special application in dislocations. It has certainly peculiar merits here, as the will is so nearly subjugated by it as to render the patient quite powerless to resist your effort at replacing, and at the same time the pain is subdued.

It is not necessary I should further continue special applications; when its *modus operandi* is understood, its adaption to many contingencies will of a sequence follow.

It is well just here, before passing to the next point of consideration, to answer a query which may arise at this juncture:

What are the successive stages of effects upon the economy from its commencement until the full effect is observed, and what proof have I that it was due to the amount of air inhaled?

The heart's action is not increased more than from seventy (the average) to eighty and sometimes ninety, but is much enfeebled, or throwing a lesser quantity of blood. The face becomes suffused, as in blowing a fire or in stooping, which continues until the breathing is suspended, when the face becomes paler. (Have not noticed any purple as from asphyxia by a deprivation of oxygen.) The vision becomes darkened, and a giddiness soon appears. The voluntary muscles furthest from the heart seem first to be affected, and the feet and hands, particularly the latter, have a numbness at their extremities, which increases, until in many cases there is partial paralysis as far as the elbow, while the limbs become fixed. The hands are so thoroughly affected that when open, the patient is powerless to close them and *vice versa*. There is a vacant gaze from the eyes and looking into space without blinking of the eyelids for a minute or more. The head seems incapable of being held erect, and there is no movement of the arms or legs as is usual when in great pain. There is no disposition on the part of the patient to take hold of the operator's hand or interfere with the operation.

Many go on breathing mechanically after the tooth has been removed, as if nothing had occurred. Some are aware that the tooth has been extracted, and say they felt it; others could not tell what had been accomplished. The majority of cases have an idea of what is being done, but are powerless to resist

With the very intelligent, or those who stop to reason, I have to teach them the peculiarities of being sensible of touch and not of pain.

One very interesting case I will state. In extracting seven teeth for a lady who was very *unwilling* to believe my statement as to touch and no pain, I first removed three teeth after having inhaled for one minute, and when fully herself, she stated that she could not understand why there was no pain while she was conscious of each one extracted; it was preposterous to believe such an effect could be possible, as her reason told her that there is connected with tooth extracting, pain in the part, and of severe character, admitting, though, she felt no pain. She allowed one to be removed without anything, and she could easily distinguish the change, and exclaimed, "It is all the difference imaginable!" When the other three were extracted, there was perfect success again as with the first three.

One of the most marked proofs of the effects of rapid breathing was that of a boy of eleven years of age for whom I had to extract the upper and lower first permanent molars on each side. He breathed for nearly a minute, when I removed in about twenty seconds all four of the teeth, without a moment's intermission or stopping the vigorous breathing; and not a murmer, sigh, or tear afterward.

He declared there was no pain, and we needed no such assertion, for there was not the first manifestation from him that he was undergoing such a severe operation.

Another case, the same day, when I had to extract the superior wisdom teeth on both sides for an intelligent young lady of eighteen years, where I had to use two pairs of forceps on each tooth (equivalent to extraction of four teeth), she was so profoundly affected afterward that she could not tell me what had been done other than that I had touched her four times. She was overcome from its effects for at least a minute afterward. She was delighted.

With such severe tests I fear very little the result in any case where I can have them do as I bid.

There can be no mistake that there is a *specific action* from something. It cannot be personal magnetism or mesmeric influence exerted by me, for such cases are rare, averaging about 10 per cent. only of all classes. Besides, in mesmeric influence the time has much to do with it; whereas, in my cases, it cannot last over a half minute or minute at most. It cannot be fear, as such cases are generally more apt to be hurt the worse. It is not diversion of mind alone, as we have an effect above it.

There is no better way of testing whether pain has been felt than by taking the lacerated or contused gums of the patient between the index finger and thumb and making a gentle pressure to collapse the alveolar borders: invariably, they they will cry out lustily, *that is pain!* This gives undoubted proof of a specific agent. There is no attempt upon my *own* part to exert any influence over my patients in any way other than that they shall believe what I say in regard to *giving* them *no pain* and in the following of my orders. Any one who knows how persons become mesmerized can attest that it is not the *operator who forces them under against their will,* but it is a peculiar state into which any who have within themselves this temperament can *place* themselves where any one who knows how can have control. It is not the will of the operator. I therefore dismiss this as unworthy of consideration in connection with rapid breathing.

Then you may now ask, To what do I attribute this very singular phenomenon?

Any one who followed, in the earlier part of this paper, the course of the argument in my soliloquy, after twenty years had elapsed from my observation upon myself of the analgesia effects of chloroform, can almost give something of an answer.

That you may the more easily grasp what I shall say, I will ask you, If it be possible for any human being to make one hundred inhalations in a minute and the heart's action is not increased more than ten or twenty pulsations over the normal, what should be the effect upon the brain and nerve centers?

If the function of oxygen in common air is to set free in the blood, either in the capillaries alone, or throughout the whole of the arterial circulation, carbonic acid gas; and that it cannot escape from the system unless it do so in the lungs as it passes in the general current—except a trace that is removed by the skin and kidneys—and that the quantity of carbonic acid gas set free is in exact relation to the amount of oxygen taken into the blood, what effect *must be* manifested where one hundred respirations in one minute are made—five or six times the normal number—while the heart is only propelling the blood a very little faster through the lungs, and *more feebly*—say 90 pulsations at most, when to be in proportion it should be 400 to 100 respirations to sustain life any length of time?

You cannot deny the fact that a definite amount of oxygen can be absorbed and is absorbed as fast as it is carried into the lungs, even if there be one hundred respirations to the minute, while the pulsations of the heart are only ninety! Nature has *made it* possible to breathe so rapidly to meet any emergency; and we can well see its beautiful application in the normal action of both the heart and lungs while one is violently running.

What would result, and that very speedily, were the act of respiration to remain at the standard—say 18 or 20—when the heart is in violent action from this running? Asphyxia would surely end the matter! And why? The excessive exercise of the whole body is setting free from the tissues such an amount of excretive matter, and carbon more largely than all the others, that, without a relative action of the lungs to admit the air that oxygen may be absorbed, carbonic acid gas cannot be liberated through the lungs as fast as the waste carbon of the overworked tissues is being made by disassimilation from this excess of respiration.

You are already aware how small a quantity of carbonic acid in excess in the air will seriously affect life. Even 2 to 3 per cent. in a short time will prove fatal. In ordinary respiration of 20 to the minute the average of carbonic acid exhaled is 4·35.

From experiments made long ago by Vierordt—see Carpenter, 524—you will see the relative per cent. of carbonic acid exhaled from a given number of respirations. When he was breathing six times per minute, 5·5 per cent. of the exhaled air was carbonic acid; twelve times, 4·2; twenty-four times, 3·3; forty-eight times, 3; ninety-six times, 2.6.

Remember this is based upon the whole number of respirations in the minute and not each exhalation—which latter could not be measured by the most minute method.

Let us deduct the minimum amount, 2·6 per cent. of carbonic acid when breathing ninety-six times per minute, from the average, at twenty per minute, or the normal standard, which is recorded in Carpenter, p. 524, as 4·35 per minute, and we have retained in the circulation nearly 2 per cent. of carbonic acid; that, at the average, would have passed off through the lungs without any obstruction, and life equalized; but, not having been thrown off as fast as it should have been, it must, of necessity, be left to prey upon the brain and nerve centres; and as 2 to 3 per cent. we are told, will so poison the blood, life is imperilled and that speedily.

It is not necessary we should argue the point as to whether oxygen displaces carbonic acid in the tissues proper or the capillaries. The theory of Lavoisier on this point has been accepted.

We know furthermore, as more positive, that tissues placed in an atmosphere of oxygen will set free carbonic acid, and that carbonic acid has a paralyzing effect upon the human hand held in it for a short time. The direct and speedy effects of this acid upon the delicate nervous element of the brain is so well known that it must be accepted as law. One of the most marked effects is the suspension of locomotion of the legs and arms, and the direct loss of will power which must supervene before voluntary muscular inactivity, which amounts to partial paralysis in the hands or feet, or peripheral extremities of the same.

Now that we have sufficient evidence from the authorities that carbonic acid can be retained in the blood by excessive breathing, and enough to seriously affect the brain, and what its effects are when taken directly into the lungs in excess, we can enter upon what I have held as the most reasonable theory of the phenomenon produced by rapid breathing for analgesic purposes; which *theory* was not *first* conceived and the process made to yield to it, but the phenomenon was long observed, and from the repetition of the effects and their close relationship to that of carbonic acid on the economy, with the many experiments performed upon myself, I am convinced that what I shall now state will be found to substantiate my discovery. Should it not be found to coincide with what some may say is physiological truth, it will not invalidate the discovery itself; for of that I am far more positive than Harvey was of the discovery of the circulation of the blood; or Galileo of the spherical shape of the earth. And I ask that it shall not be judged by my theory, but from the practice.

It should have as much chance for investigation as the theory of Jullus Robert Meyer, upon which he founded, or which gave rise to the establishment of one of the most important scientific truths—"the conservation of energy," and finally the "correlation of forces," which theory I am not quite sure was correct, although it was accepted, and as yet I have not seen it questioned.

With all due respect to him I quote it from the sketch of that remarkable man, as given in the *Popular Science Monthly*, as specially bearing on my discovery:

"Mayer observed while living in Java, that the *venous blood* of some of his patients had a singularly bright red color. The observation riveted his attention; he reasoned upon it, and came to the conclusion that the brightness of the color was due to the fact that a less amount of oxidation was sufficient to keep up the temperature of the body in a hot climate than a cold one. The darkness of the venous blood he regarded as the visible sign of the energy of the oxidation."

My observation leads me to the contrary, that the higher the temperature the more rapid the breathing to get clear of the excess of carbon, and hence more oxygenation of the blood which will arterialize the venous blood, unless there is a large amount of carbonized matter from the tissues to be taken up.

Nor must it be denied because of the reasoning as presented to my mind by some outside influence in my soliloquy, when I first exclaimed, " Nature's anæsthetic," where the argument as to the effects of nitrous oxide gas being due to an excess of oxygen was urged, and that common air breathed in excess would do the same thing.

I am not sure that *it* was correct, for the effect of nitrous oxide is, perhaps, due to a deprivation of mechanically mixed air.

Knowing what I do of theory and practice, I can say with assurance that there is not a medical practitioner who would long ponder in any urgent case as to the thousand and one theories of the action of remedies, but would resort to the *practical* experience of others and his own finally. (What surgeon ever stops to ask how narcotics effect their influence?) After nearly thirty years of association with ether and chloroform, who can positively answer as to their *modus operandi?* It is thus with nearly the whole domain of medicine. It is not yet, by far, among the sciences, with immutable laws, such as we have in chemistry. Experimentation is giving us more specific knowledge, and "practice alone has tended to make perfect." Then, gentlemen will not set at naught my assertion and practical results. When I have stated my case in full it is for *you* to disprove both the theory and practice annunciated. So far as I am concerned I am responsible for both.

You will please bear with me for a few minutes in my attempt at theory.

The annulling of pain, and, in some cases, its complete annihilation, can be accomplished in many ways. Narcotics, anæsthetics—local and internal—direct action of cold, and mesmeric or psychological influence, have all their advocates, and each *will surely* do its work. There is one thing about which, I think we can all agree, as to these agencies: unlesss the *will* is partially and in some cases completely subjugated there can be primary or secondary effect. The voluntary muscles must become wholly or partially paralyzed for the time. Telegraphic communication must be cut off from the brain, that there be no reflex action. It is not necessary there should be separate nerves to convey pleasure and pain any more than there should be two telegraphic wires to convey two messages.

If, then, we are certain of this, it matters little as to whether it was done by corpuscular poisoning and anæmia as from chloroform or hyperæmia from ether.

I think we are now prepared to show clearly the causes which effect the phenomena in "rapid breathing."

The first thing enlisted is the *diversion of the will force* in the act of forced respiration at a moment when the heart and lungs have been in normal reciprocal actian (20 respirations to 80 pulsations), which act could not be made and carried up to 100 respirations per minute without such concentrated effort that ordinary pain could make no impression upon the brain while this abstraction is kept up.

Second. There is a specific effect resulting from enforced respirations of 100 to the minute, due to the *excess of carbonic acid gas set free from the tissues*, generated by this enforced normal act of throwing into the lungs *five times* the normal amount of oxygen demanded in one minute, when the heart has not been aroused to exalted action, which comes from violent exercise in running or where one is suddenly startled, which excess of carbonic acid cannot escape in the same ratio from the lungs, since the heart does not respond to the proportionate overaction of the lungs.

Third. Hyperæmia is the last in this chain of effects, which is due to the excessive amount of air passing into the lungs preventing but little more than the normal quantity of blood from passing from the heart into the arterial circulation, but damming it up in the brain with its excess of carbonic acid gas to act also directly upon the brain as well as throughout the capillary and venous system, and as well upon the heart, the same as if it were suspended in that gas outside the body.

These are evident to the senses of any liberal observer who can witness a subject rapidly breathing.

Some ask why is not this same thing produced when one has been running rapidly for a few minutes? For a very good reason: in this case the rapid inhalations are preceded by the violent throes of the heart to propel the carbonized blood from the overworked tissues and have them set free at the lungs where the air is rushing in at the normal ratio of four to one. This is not an abnormal action, but is of necessity, or asphyxia would instantly result and the runner drop. Such sometimes occurs where the runner exerts himself too violently at the very outset; and to do so he is compelled to hold his breath for this undue effort, and the heart cannot carry the blood fast enough. In this instance there is an approach to analgesia as from rapid breathing.

Let me take up the first factor—*diversion of will*—and show that nature invariably resorts to a sudden inhalation to prevent severe infliction of pain being felt. It is the panacea to childhood's frequent bruises and cuts, and every one will remember how when a finger has been hurt it is thrust into the mouth and a violent number of efforts at rapid inhalation is effected until ease comes. By others it is subdued by a fit of crying, which if you will but imitate the sobs, will find how frequently the respirations are made.

One is startled, and the heart would seem to jump out of the chest; in quick obedience to nature the person is found making a number of quick inhalations, which subdue the heart and pacify the will by diversion from the cause.

The same thing is observed in the lower animals. I will relate a case:

An elephant had been operated upon for a diseased eye which gave him great pain for which he was unprepared, and he was wrathy at the keeper and surgeon. It soon passed off, and the result of the application was so beneficial to the animal that when brought out in a few days after, to have another touch of caustic to the part, he was prepared for it; and just before the touch, he inflated the lungs to their fullest extent, which occupied more time than the effect of the caustic, when he made no effort at resistance and showed no manifestation of having been pained.

In many cases of extraction of the temporary teeth of children, I make them at the instant I grasp the tooth take *one* very violent inhalation, which is sufficient. Mesmeric anæsthesia can well be classified under diversion or subjugation of the will, but can be effected in but a small percentage of the cases. To rely upon this first or primary effect, except in instantaneous cases, would be failure.

The second factor is the one upon which I can rely in such of the cases as come into my care, save when I cannot induce them to make such a number of respirations as is absolutely necessary. The *whole secret of success lies* in the greatest number of respirations that can be effected in from 60 to 90 seconds, and that without any intermission. If the heart, by the *slow method of respiration*, is pulsating in ratio of four to one respiration, *no effect can be induced.*

When the respirations are, say, 100 to the minute, and made with all the energy the patient can muster, and are kept up while the operation is going on, there can hardly be a failure in the minor operations.

It is upon this point many of you may question the facts. Before I tried it for the first time upon my own person, I arrived at the same conclusion from a course of argument, that rapid breathing would control the heart's action and pacify it, and even reduce it below the normal standard, under my urgent respirations.

In view of the many applications made, I feel quite sure in my belief that, inasmuch as the heart's action is but slightly accelerated, though with less force from rapid breathing at the rate of 100 to the minute, there is such an excess of carbonic acid gas set free and crowding upon the heart and capillaries of the brain, without a chance to escape by the lungs, that it is the same to all intents as were carbonic acid breathed through the lungs in common air. Look at the result after this has been kept up for a minute or more. During the next minute the respirations are not more than one or two, and the heart has fallen really below, in some cases, the standard beat, showing most conclusively that once oxygenation has taken place, and that the free carbonic acid gas has been so completely consumed, there is no involuntary call through the pneumogastric nerve for a supply of oxygen.

If any physiological facts can be proven at all, then I feel quite sure of your verdict upon my side.

There is one thing that goes so far to prove the theory of Lavoisier regarding the action of oxygen in the tissues and capillaries for converting carbon into carbonic acid gas instead of the lungs, as held prior to that time, and still held by many who are not posted in late experiments. At the time I commenced this practice I must confess I knew nothing of it. The study of my cases soon led me to the same theory of Lavoisier, as I could not make the phenomena agree with the old theory of carbonic acid generated only in the lungs.

When Vierordt was performing his experiments upon himself in rapid breathing from six times per minute to ninety-six, I cannot understand why he failed to observe and record what did certainly result—an extreme giddiness with muscular prostration and numbness of the peripheries of the hands and feet, with suffusion of the face, and such a loss of locomotion as to prevent standing erect without desiring support. Besides, the very great difference he found in the amount of carbonic acid retained in the circulation, the very cause of the phenomena just spoken of.

One thing comes in just here to account for the lack of respiration the minute after the violent effort. The residual air, which in a normal state is largely charged with carbonic acid, has been so completely exhausted that some moments are consumed before there is sufficient again to call upon the will for its discharge.

As to hyperæmia, you will also assent, now that my second factor is explained; but it is so nearly allied to the direct effect of excessive respiration that we can well permit it to pass without argument. If hyperœmia *is present*, we have a more certain and rather more lasting effect.

In conclusion, I will attempt to prognosticate the application of this principle to the cure of many diseases of chronic nature, and especially tuberculosis; where from a diminished amount of air going into the lungs for want of capacity, and particularly for want of energy and inclination to breathe in full or excess, the tissues cannot get clear of their excrementitious material, and particularly the carbon, which must go to the lungs, this voluntary effort can be made frequently during the day to free the tissues and enable them to take nutritious material for their restoration to their standard of health.

Air will be found of far more value than ever before as one of the greatest of factors in nutrition, and which is as necessary as proper food, and without which every organization must become diseased, and no true assimilation can take place without a due amount of oxygen is hourly and daily supplied by this extra aid of volition which has been so long overlooked.

The pure oxygen treatment has certainly performed many cures; yet, when compared to the mechanical mixture and under the direct control of the will, at all times and seasons, there is no danger from excessive oxygenation as where pure oxygen is given. When every patient can be taught to rely upon this great safety valve of nature, there will be less need for medication, and the longevity of our race will be increased with but little dread by mankind of that terrible monster consumption, which seems to have now unbounded control.

When this theory I have here given you to-night is fully comprehended by the medical world and taught the public, together with the kind of foods necessary for every one in their respective occupation, location, and climate, we may expect a vast change in their physical condition and a hope for the future which will brighten as time advances.

The following incident which has but very recently been made known, gives most conclusive evidence of the truth of the theory and practice of rapid breathing.

A Mexican went into the office of a dentist in one of the Mexican cities to have a tooth extracted by nitrous oxide gas.

The dentist was not in, and the assistant was about to permit the patient to leave without removing the tooth, when the wife of the proprietor exclaimed that she had often assisted her husband in giving the gas, and that she would do so in this instance if the assistant would extract the tooth. It was agreed. All being in readiness, the lady turned on, as she supposed, the gas, and the Mexican patient was ordered to breathe as fast as possible to make sure of the full effect and no doubt of the final success. The assistant was about to extract, but the wife insisted on his breathing more rapidly, whereupon the patient was observed to become very dark or purple in the face, which satisfied the lady that the full effect was manifested, and the tooth was extracted, to the great satisfaction of all concerned. While the gas was being taken by the Mexican the gasometer was noticed to rise higher and higher as the patient breathed faster, and not to sink as was usual when the gas had been previously administered. This led to an investigation of the reason of such an anomalous result, when to their utter surprise they found the valve was so turned by the wife that the Mexican had been breathing nothing but common air, and instead of exhaling into the surrounding air, he violently forced it into the gasometer with the nitrous oxide gas, causing it to rise and not sink, which it should have done had the valve been properly turned for the passage of gas into the lungs of the patient.

No more beautiful and positive trial could happen, and might not by accident or inadvertence happen again in a lifetime. A similar case was related to me, by the dentist in charge, which happened in Arlington, Del., but he could not, as in the case of the Mexican, understand why it was so.—*Scientific American Supplement.*

